# A framework for tracer-based metabolism in mammalian cells by NMR

**DOI:** 10.1038/s41598-018-37525-3

**Published:** 2019-02-21

**Authors:** Raquel Saborano, Zuhal Eraslan, Jennie Roberts, Farhat L. Khanim, Patricia F. Lalor, Michelle A. C. Reed, Ulrich L. Günther

**Affiliations:** 1 University of Birmingham, Institute of Cancer and Genomic Sciences, Birmingham, B15 2TT England; 2 University of Birmingham, School of Biosciences, Birmingham, B15 2TT England; 3 University of Birmingham, Institute of Immunology and Immunotherapy, Birmingham, B15 2TT England

## Abstract

Metabolism changes extensively during the normal proliferation and differentiation of mammalian cells, and in cancer and inflammatory diseases. Since changes in the metabolic network reflect interactions between genetic, epigenetic and environmental changes, it is helpful to study the flow of label from isotopically labelled precursors into other metabolites rather than static metabolite levels. For this Nuclear Magnetic Resonance (NMR) spectroscopy is an attractive technique as it can quantify site-specific label incorporation. However, for applications using human cells and cell lines, the challenge is to optimize the process to maximize sensitivity and reproducibility. Here we present a new framework to analyze metabolism in mammalian cell lines and primary cells, covering the workflow from the preparation of cells to the acquisition and analysis of NMR spectra. We have applied this new approach in hematological and liver cancer cell lines and confirm the feasibility of tracer-based metabolism in primary liver cells.

## Introduction

The metabolism of a cell changes as a net downstream response to the cellular environment. This is triggered by external stimuli influencing the cell cycle and the balance between differentiation, proliferation and apoptosis, which all contribute to changes in metabolic networks. Furthermore, genetic and epigenetic changes also modify metabolism and the metabolic response to extrinsic factors. Thus, in order to better understand the regulation of metabolism, one needs to integrate analysis of regulatory protein levels with a quantitative analysis of metabolite levels.

Since the seminal findings by Otto Warburg of increased glycolysis leading to lactic acid production in cancer cells, it has become increasingly common to use metabolomic approaches to quantify levels of key mediators within cells^1^. However, quantification of metabolite concentration provides only a static picture of metabolic processes that are in constant flux. In order to elucidate metabolic mechanisms in cells one needs to employ metabolic flux analysis, or at least tracer-based metabolism. The advantage of tracer-based analyses is that changes in metabolites can be assigned to particular mechanisms. For tracer-based analyses, different isotopically labelled precursors have been used as starting points to determine the intermediates and products of metabolism in cells. Typically-used isotopes include ^13^C and ^15^N, both are amenable to NMR examination. A tracer-based metabolic analysis can assign a product to one particular or multiple pathways, or even describe the contribution of different pathways which is often not possible based on static metabolite concentrations. A typical example is the consumption and/or production of glutamic acid in cancer cells, which can involve production from glycolysis and the Krebs cycle or from glutamine^2^. Tracer-based methods can also distinguish between different entry mechanisms into the Krebs cycle, to determine contributions of pyruvate dehydrogenase activity vs the anaplerotic pathways using pyruvate carboxylase or glutaminolysis. In the past ^13^C-labelled precursors such as glucose, glutamine, glutamic acid, pyruvate, acetate, aspartate, glycerol, serine and fatty acids^3^ have been used for this purpose.

The two predominantly used technologies in this context have been mass spectrometry (MS) and NMR spectroscopy. MS has a significant advantage over NMR in sensitivity. However, its information content is limited to mass increments, which can often only be interpreted in the context of predefined models^4,5^. With the mechanistic details we now see unraveled in current biology, it is becoming increasingly clear that it is desirable to add an analytical level that can also detect site-specific label incorporation in small molecules.

First applications of NMR for tracer-based metabolism reach back into the 1970s^6–8^, with significant progress in the 1990s when Szyperski and Wüthrich^9–11^ introduced ^1^H-^13^C-HSQC spectra for such analyses. Chikayama has used HSQC spectra and other spectra to assign metabolites in plants and silkworm larvae^12^. Several seminal publications have established roadmaps for tracer-based metabolism using mass spectrometry, in particular in the context of metabolic flux analysis^13,14^.

Here we present a workflow for efficiently using NMR in the context of tracer-based metabolism using mammalian cell lines or even primary cells under physiologically relevant conditions. This includes methods of preparing cells, along with NMR methods suitable for such analyses. We also discuss possible precursors that can be used to decipher different pathways. Furthermore, we show applications in cancer cell lines and in primary liver cells.

## Results

### The tracer-based metabolism framework

**Figure 1:**
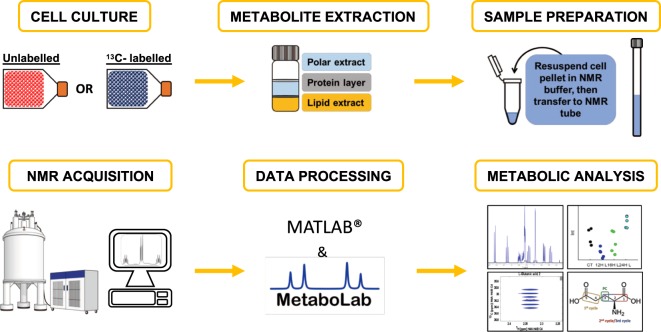
Workflow for NMR tracer-based metabolism. Cell cultures are grown in a medium containing ^13^C or ^15^N- labelled precursors. Typically, 10–20 million cells are required. Adherent cells are removed from the flask surface using a scraper, thereby avoiding the use of trypsin which generates unwanted metabolites. Cells in suspension (e.g. hematological cancer cells) are harvested by centrifugation (See Materials and Methods for detailed protocols). Metabolites are obtained using a methanol/chloroform/water system where the polar extract in the upper phase is easily collected. Samples are vacuum dried and re-suspended in NMR buffer before being transferred to an NMR tube. Different NMR spectra are acquired, typically including a 1D-^1^H-NOESY and a 2D-^1^H,^13^C-HSQC, often also 2D-^1^H,^1^H-TOCSY spectra. For a complete isotopomer analysis, a combination of NMR and GC-MS may be used, which can be analyzed using the MetaboLab software^24^. From this analysis site specific label incorporations can be derived^19^. The spectrometer in this Figure is taken from WikiCommons, CC BY-SA 3.0 (https://creativecommons.org/licenses/by-sa/3.0/) https://commons.m.wikimedia.org/wiki/File:Nuclear_magnetic_resonance_(NMR).png.

Figure 1 shows the workflow that we propose for NMR tracer-based metabolism. The different stages of this process will be discussed below, with an emphasis on steps taken to increase reproducibility between samples, to maximize sensitivity per unit time in the NMR experiments, and to obtain validated results.

### NMR Sample Preparation

**Figure 2:**
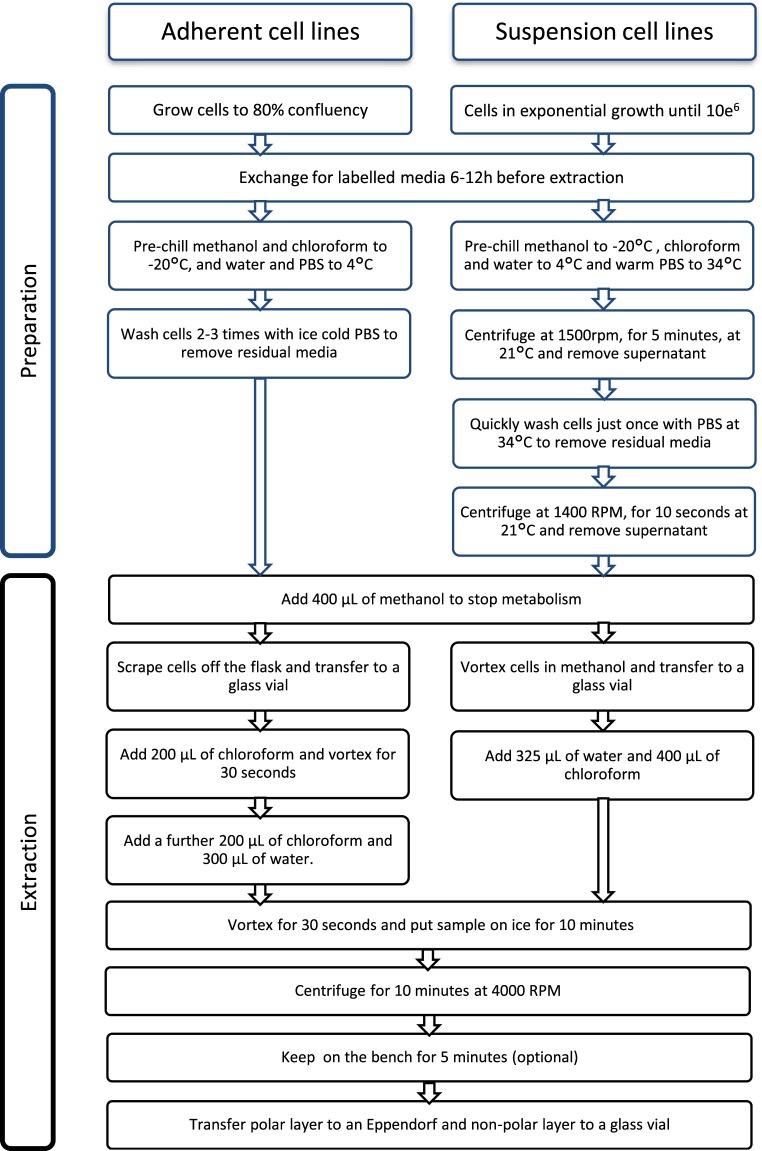
Flowchart summarizing the preparation of cells for tracer-based metabolism NMR analyses for adherent cell lines (left) and for cells growing in suspension (right).

Reproducible metabolite extraction from cell lines or primary cells is not trivial as metabolism is not static and metabolite concentrations change quickly, even during the extraction process. High reproducibility is of particular importance for comparing labeled and unlabeled samples. Cells must be grown within a controlled environment, with identical cell numbers, and typically a final cell number of 10–20 million cells must be obtained in order to acquire ^1^H,^13^C-HSQC and other NMR spectra in a reasonable time frame. Highly reproducible growth conditions are required and the amount of media must be minimized as labeled precursors are expensive. The overall procedure is summarized in the flowchart depicted in Fig. 2 with further details provided in Materials and Methods. To achieve optimal reproducibility, it is essential that cold solvents are used for the extraction to stop all metabolic processes^15^. We chose to lyse the cells using cold methanol which has the advantage of immediately quenching metabolism, followed by chloroform extraction to obtain and separate the organic and polar phase. Alternatively, others have used liquid nitrogen to quench metabolism or to lyse cells by sonication. The latter has the drawback of long incubation times, requiring enzyme inhibitors to stop protein degradation, but allows the fractionation of cell compartments^16,17^. Media for tracer-based metabolism can either be prepared from scratch or media with one or several metabolites missing can be prepared or purchased. For any quantitative analysis it is important to replace the entire pool of one or several metabolites with its isotope labelled equivalent.

**Figure 3:**
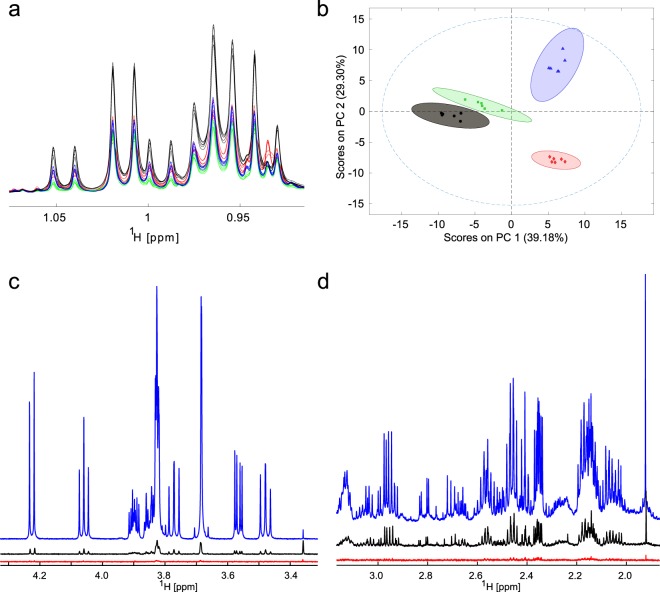
Increasing the power of NMR metabolomics analysis by increasing sample reproducibility, and by enhancing sensitivity with modern probe technology. (**a**) Spectra of four hematological cell lines (black: U266 and green: UM3, both myeloma cell lines, blue: Sudhl6 diffuse large B-cell lymphoma, red: Glor Burkitt’s lymphoma cell line) with 6 replicates per cell line to illustrate the required level of reproducibility. (**b**) Principal Component Analysis plot of spectra shown in (**a**) with 95% confidence intervals showing a clear separation between classes of cells and excellent clustering within the individual cell lines. (**c**,**d**) Comparison of 1D spectra obtained from two equal mass samples, either sucrose (**c**) or hepatocarcinoma cell line (HuH7) extracts **(d)** using a 1.7 mm micro-cryoprobe (blue), a 5 mm cryoprobe (black) and a 5 mm room temperature (RT) probe (red). These panels illustrate a factor 5–7 sensitivity gain from the RT to the cryoprobe, and another factor of 5–7 for the micro-cryoprobe. Overall the gain for equal sample masses from the RT to the micro-cryoprobe can be up to 40 fold.

When this protocol is followed rigorously we obtain 1D-NMR spectra with excellent reproducibility, as shown in Fig. 3a for a series of hematological cancer cell lines. The variability between intensities within replicates should be between 1 and 3% as relevant changes in metabolism can be small. Data of this quality enables excellent separation of different cell-lines by principal component analysis (PCA) (Fig. 3b). Without this level of reproducibility, it is impossible to compare labelled and unlabeled spectra in a quantitative analysis, and comparing metabolism between different samples also becomes questionable.

### Improvements in NMR technology

Tracer-based NMR analysis is considerably facilitated by huge signal-to-noise improvements arising from the introduction of micro-cryoprobe technology by the Bruker company. This is illustrated by comparing spectra from equal mass samples acquired with a 5.0 mm room temperature (RT) probe, a 5.0 mm cryoprobe and a 1.7 mm micro-cryoprobe (Fig. 3c,d). For this assessment, sucrose samples were prepared to a final concentration of 1 mM when using the micro-cryoprobe, i.e. 12 µg of sucrose in 35 µL for the micro-cryoprobe and in 600 µL for the other probes. We observed essentially no signal after 32 scans in the RT probe and a S/N ratio gain of at least 10 from using the 1.7 mm cryoprobe instead of the 5 mm cryoprobe. The same trend is illustrated for cell extracts from hepatocarcinoma cell line (HuH7, Fig. 3d). Two samples were prepared from the same flask. To ensure that equal cell masses were used, we vacuum-dried equal volumes from the polar layer of the methanol/chloroform/water extraction and dissolved these samples in different volumes as explained previously. Here the RT probe shows very low-intensity signals for 128 scans, whereas cryoprobes show a huge improvement with a further signal enhancement factor of 4–5 for the micro-cryoprobe.

Overall sensitivity improves by a factor of at least 40 with this probe technology for both samples. Importantly, the salt tolerance of this type of probe is much better than that of conventional cryoprobes but shimming becomes more challenging due to the large length/width ratio and the temperature gradient along the sample length of the NMR tube.

### Tailoring HSQC experiments for tracer-based studies

It is primarily this advancement in technology that enables the use of 2D-^1^H,^13^C-HSQC spectra from cell extracts within a reasonable time period of 4 to 5 hours. HSQC spectra represent one of several choices for this type of analysis. By correlating proton (^1^H) and ^13^C (or ^15^N) chemical shifts, spectral overlap is considerably reduced compared with homonuclear two-dimensional (2D) experiments, such as ^1^H-^1^H-TOCSY. HSQC spectra offer at least two options for the measurement of label incorporation: (i) Measuring intensities from metabolite spin systems, which rise with the amount of label incorporation. This approach requires an unlabeled reference spectrum, and to derive absolute concentrations, it is also necessary to calibrate for different metabolites owing to variable coupling constants (see^18^ and *Materials and Methods*). (ii) Analysis of the complex multiplet patterns in the indirect ^13^C-dimension arising from scalar couplings (^1^*J*_CC_ couplings) between adjacent ^13^C-atoms. This enables the determination of label incorporations in adjacent atoms as shown recently^19^.

Using signal intensities in HSQC spectra as analytical tools to quantify site-specific label incorporation ratios in metabolites requires appropriate scaling. This is challenging as algorithms typically used for 1D spectra fail because intensity depends on label incorporation levels, as well as concentration (The intensity of HSQC signals is proportional to I_HSQC_ ∼ *Γ* · n · x, where *Γ* is the pulse sequence specific transfer function, n the number of spins, and x the label incorporation). In our workflow we have used the total spectral area (TSA) or Probabilistic Quotient Normalization (PQN)^20^ in the corresponding 1D spectrum of the same sample to scale the HSQC (see Materials and Methods for further details). These methods fail in the presence of systematic changes affecting many metabolites. In such cases it is sometimes possible to use intensities of selected metabolites, e.g, the sum of all branched chain amino acids, as a reference. For well-resolved resonances the accuracy of label incorporations may be cross-checked using ^13^C-edited and filtered spectra.

To resolve *J*_CC_ coupling constants high-resolution HSQC spectra are needed, typically acquired with 16384 increments but only 2 scans. To acquire such spectra, we use non-uniform sampling (NUS) with a 25% sampling rate. Such spectra can be acquired in approximately 4.5 h. The multiplet patterns obtained this way are virtually indistinguishable from those with regular sampling (Supplementary Fig. 2). Spectra acquired using NUS can be processed using different approaches^21,22^ and the application for small molecules has recently been demonstrated^23^.

It is important to realize that signals in HSQC spectra arise solely from protons attached to ^13^C. In contrast to 1D-^1^H and ^1^H, ^1^H-TOCSY spectra, a ^1^H-^12^C pair does not lead to any signal in an HSQC spectrum. In mass spectrometry terms, it is only the m ≥ +1 molecules that can be observed. In other words, the interpretation of HSQC spectra is complicated by the fact that the amount of ^12^C for a particular atom in a metabolite cannot be measured directly. In principle, one can estimate the amount of unlabeled sample from intensities in the natural abundance spectrum; the total amount of carbon (^13^C + ^12^C) must then be 100 times the observed ^13^C. For a labeled sample grown under identical conditions, the amount of the metabolite will be same, although different isotopomers may be present. The level of label incorporation can be inferred by comparing signal intensities in the labelled and natural abundance reference spectra.

Although signal intensities arising from the 1.1% natural abundance ^13^C in most metabolites can be quite low, which limits quantification, comparing relative intensities between related labelled samples can help overcome this problem. In this paper we demonstrate how intensities from both unlabeled and labeled HSQC metabolites can be used to determine the ^13^C-incorporation in specific positions.

The value of using HSQC spectra for isotopomer analysis arises from the quantitative information in the multiplet structure in the incremented ^13^C-dimension, as originally suggested by Szyperski^10^. In a mixture of isotopomers for the central carbon of a 3-carbon fragment, there is a superimposition of resonances arising from isolated ^13^C singlets, two possible ^13^C-^13^C doublets, and the possibility of doublets of doublets when all three carbons are labelled (Supplementary Fig. 4). The MetaboLab software^24^ has a module that simulates line shapes and deconvolutes contributions from different isotopomers in slices taken from HSQC spectra^19^.

**Figure 4:**
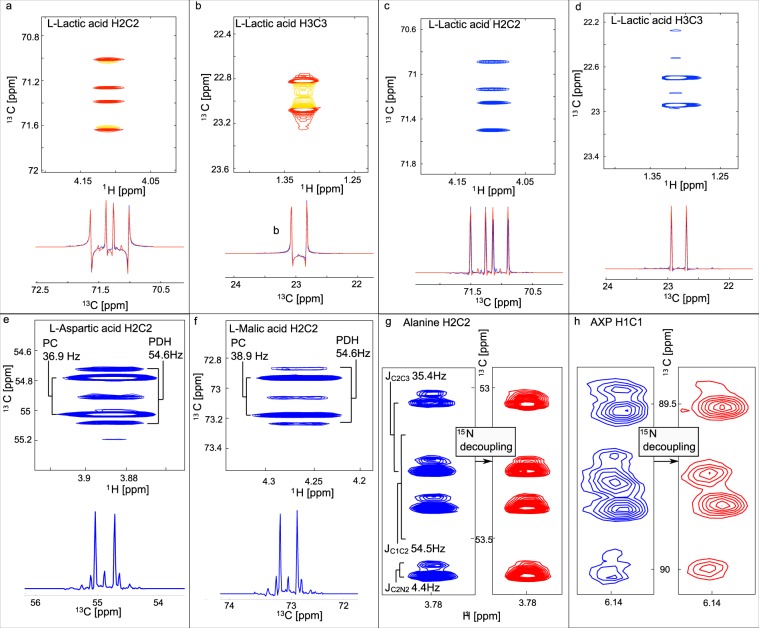
Sections from ^1^H-^13^C HSQC spectra of polar cell extracts illustrating spectral improvements obtained using acquisition mode of the indirect directions States rather than Echo-AntiEcho in HSQCs with NUS. (**a**–**d**) Show slices and spectral regions for L-lactic acid resonances from ^1^H,^13^C-HSQC spectra for HuH7 cells grown for 12 hours in a medium containing [U-^13^C] glucose. Echo-antiecho-^1^H,^13^C-HSQC spectra acquired for samples in a mixed 90% H_2_O/10% D_2_O NMR buffer (panels a and b) and conventional ^1^H,^13^C-HSQC spectra acquired for samples in a D_2_O buffer (panels c and d). The high resolution in the ^13^C dimension obtained with 25% NUS sampling over 8k complex points is illustrated in panels **e** and **f** where the different couplings obtained from the PC-derived ^13^C_2_–^13^C_3_ and PDH-derived ^13^C_2_-^13^C_1_ moieties are clearly resolved in the L-aspartic acid (**e**) and L-malic acid (**f**) H_2_C_2_ resonances. (**g)** shows the alanine H2/C2 resonance at 3.78/53.2ppm from GLOR cells labelled with [U-^13^C]glucose and [^15^N]glutamine. The complexity of the ^15^N-undecoupled (blue) spectrum arises from the superimposition of resonances from mainly [^13^C-]alanine with a small proportion of [^15^N,^13^C]alanine. While *J*_C2C3_ and *J*_C1C2_ produce a well-resolved doublet of doublets. For [^15^N,^13^C]alanine, these 4 resonances are further split by the small *J*_C2N2_ coupling. The resulting spectrum is further complicated by a ^14^N/^15^N isotope shift that causes overlap of one of the double components of [^13^C,^15^N]alanine with the resonance from [^13^C]alanine. (**f):** AXP (ADP and AMP) H_1_/C_1_ resonances at 6.15/89.7ppm from [^15^N,^13^C]glucose and [^15^N]glutamine labelled GLOR cells showing a complex pattern based on a doublet due to the large ^1^*J*_C1C2_ coupling in the undecoupled (blue) spectrum. Additional resonances are seen because a proportion of the C1 is directly attached to a ^15^N in the adenine ring. This complexity is reduced by ^15^N decoupling in the red spectrum but the remaining peaks appear broadened by the ^14^N/^15^N isotope shift^45^ on the directly-attached ^13^C.

For such HSQC spectra, the choice of HSQC pulse sequence is important. The echo-antiecho-selected HSQC^25^ is commonly used as it provides excellent solvent and artefact suppression, but leads to complex line shapes in the presence of ^2^*J*_CC_ couplings (Fig. 4a,b). These arise from scalar coupling evolution during the time when the selective encoding gradient is applied. While such signals can be simulated using a quantum mechanical approach^19^, pure in-phase absorption type signals can be obtained when using HSQC sequences without echo-antiecho encoding (Fig. 4c,d). Such spectra are best acquired in D_2_O due to reduced solvent suppression efficiency. Simulation of lineshapes in the incremented dimension can be used for isotopomer analysis^19^. Examples of such simulations are shown in Fig. 4a–d for lactic and for glutamic acid in samples derived from HuH7 cells (blue experimental, red simulated spectra). This approach is particularly robust as it does not depend on signal intensities. In-phase spectra acquired in D_2_O offer a fast and reliable assessment of metabolic mechanisms without any need of simulations.

Additional information can also be inferred from NMR proton observed NMR spectra which show ^13^C and ^12^C- linked signals, such as 1D-^1^H or 2D-^1^H-TOCSY. This approach has been successfully employed by Fan and coworkers^26^. The limitation here lies in the overlap of signals in metabolite mixtures derived from mammalian cells. From TOCSY spectra it is usually possible to obtain label incorporations for several metabolites as shown for lactic acid and threonine. The same is possible for some other metabolites including alanine, glutamic acid, aspartate, aspartate derived UDP-pyrimidine resonances, although signal overlap is a problem for less abundant metabolites (Supporting Fig. 3). Another option is the use of ^13^C-filtered 1D-^1^H-spectra^27^ and possibly ^13^C-filtered TOCSY spectra.

### Insights from tracer-based analyses

**Figure 5:**
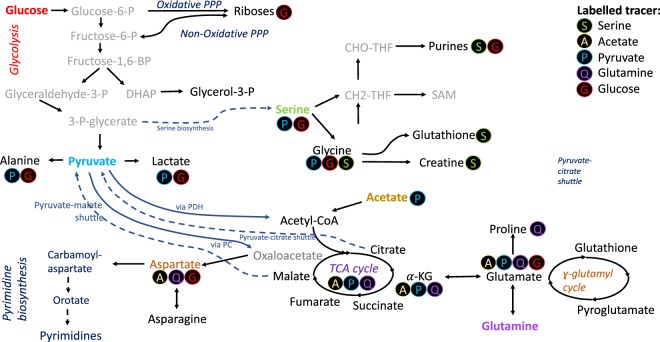
Label incorporation schemes for NMR.

Several metabolic pathways from central carbon metabolism can be explored using the aforementioned methods, as summarized in Fig. 5. These include glycolysis, the oxidative and non-oxidative branch pentose phosphate pathway (PPP), Krebs cycle metabolism, the relative importance of pyruvate dehydrogenase and pyruvate carboxylase^28^ in feeding pyruvate-derived carbons into the Krebs cycle, glutaminolysis in the Krebs cycle^2^ nucleotide synthesis, serine/glycine, creatine synthesis and one carbon metabolism (Supplementary Table 1). This list is likely to lengthen with the use of additional labeling schemes.

It has previously been shown that [1,2-^13^C]glucose and [3-^13^C]glutamine represent excellent choices for such analyses^29,30^ In many cases it is useful to have multiple carbons labeled in the same molecule as the preservation of adjacent labels bears considerable information. For example in [1,2-^13^C]glucose labelled samples, the size of the coupling constant in aspartate and malate can be used to determine the balance between pyruvate carboxylase (PC) and pyruvate dehydrogenase (PDH) activity^28^, as the two entry mechanisms for the TCA cycle produce different labelling patterns. In the case of PC C2&3 of aspartate and malate become labelled with a smaller coupling constant compared to coupling to the COO groups in the case of PDH activity (C1&2 or C3&4). An example is depicted in Fig. 4e,f which is similar to what we have previously observed, although now with considerably improved spectral quality. In haematological cancer cells we often observe that aspartate is primarily formed from PC, and in one case (in K562 cells) we were able to follow the path of label incorporation via carbamoyl-aspartate, dihydroorotate and orotate into pyrimidines^2^.

Lactate label incorporation can be used to identify PPP activity with [1,2-^13^C]glucose as a precursor because the oxidative PPP removes C1 from glucose and this alters label incorporation in lactic acid and in riboses^28^. We also found that uridine diphosphate (UDP) can be used to determine relative amounts of oxidative vs non-oxidative PPP branch activities^2^. Pyruvate is well-known as an agent for fast metabolic fluxes using Dynamic Nuclear Polarization (DNP) MRI. In this process the ^13^C1 is polarized as it has the longest *T*_1_ relaxation time. For HSQC analysis the C2,3 are more informative. Interestingly we also observed serine and glycine formation in U266 multiple myeloma cell lines. Serine is a valuable precursor as it gets catabolized into glycine and formate, and both become building blocks for pyrimidines.

#### Dual labeling

Another option with NMR is to label multiple precursors in a way that metabolite signals will show couplings arising from label incorporation in adjacent positions with isotopes derived from two different labelled precursors. For example we observe alanine with *J*_C2C3_ and *J*_C2N_ coupling arising from labelling with [U-^13^C]glucose and [^15^N]glutamine (Fig. 4g). This double labelling strategy is particularly useful to look at nucleotide synthesis (Supplementary Fig. 4). For example, the C1 of the ribose of adenosine nucleotides shows coupling to the directly attached ^15^N of the adenine ring from labelling with [U-^13^C]glucose and [^15^N]glutamine (Fig. 4h). For double labelling, we have explored several combinations: [U-^13^C]glucose and [^15^N]glutamine, and [1,2-^13^C]glucose and [3-^13^C]glutamine. These experiments facilitate the assessment of the contributions of glycolysis and glutaminolysis in feeding the Krebs cycle. Likewise, one-carbon metabolism can be probed by [^13^C,^15^N]serine and [^15^N]glutamine. A recent report shows the utility of combining [1,2-^13^C]acetate and [1,6-^13^C]glucose^31^.

### Proof of concept studies using human cells

Carbon-13 enrichment was explored using various labeled precursors for different human cell lines, including the adherent hepatocellular epithelial cell line HuH7, and two hematological cancer cell lines, U266, a multiple myeloma cell line, and GLOR, a Burkitt’s lymphoma cell line. The choice of GLOR and U266 cell lines permitted comparison of a more glycolytic cell line and a cell line with stronger Krebs cycle activity. For GLOR and U266, we compare glucose and glutamine labeling, however HuH7 cells do not seem to consume glutamine over the period of this experiment. Moreover, we looked at serine and pyruvate as precursors for hematological cell lines.

**Figure 6:**
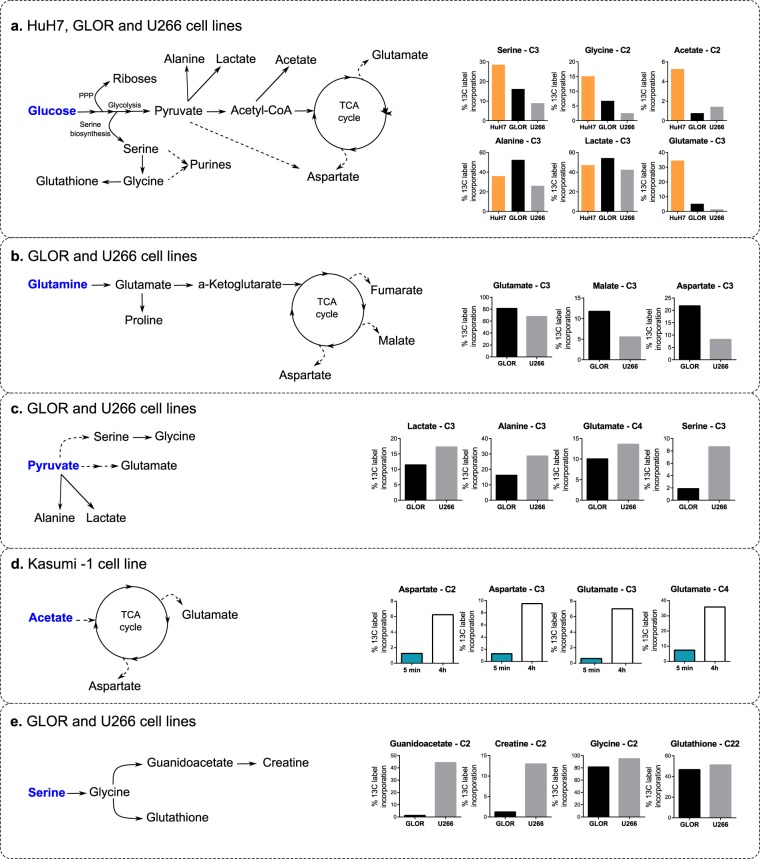
Label incorporations in extracts from different cell types. The labelled precursors used were [U-^13^C]glucose (**a**), [3-^13^C]glutamine (**b**), [3-^13^C]pyruvate (**c**) and [2-^13^C]acetate (**d**) and [^15^N,^13^C]serine (**e**). In a–c & e, bars denote label incorporations in the U266, GLOR and HuH7 cell-lines respectively (additional information in Supplementary Fig. 5).

Figure 6a,b compare label incorporations using [U-^13^C]glucose and [3-^13^C]glutamine, respectively, as precursors. GLOR show a typical Warburg effect with greater lactate and alanine production and glutaminolysis to supply the Krebs cycle, driven by c-Myc overexpression. Only the HuH7 hepatocytes produced labelled aspartate and showed labelling in aspartate-derived pyrimidine nucleotides from glucose.

**Figure 7:**
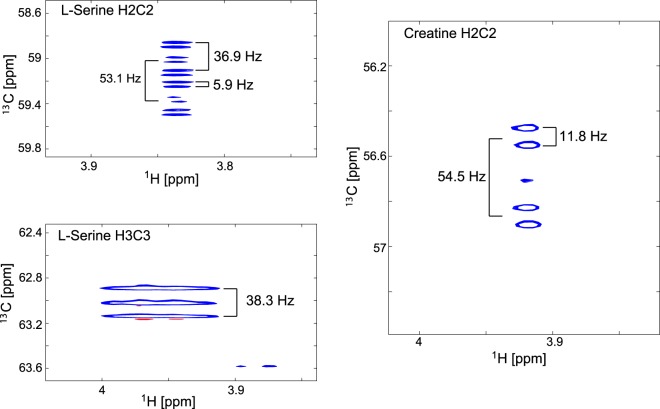
Coupling patterns at the C2/H2 resonances of serine and creatine in [U-^13^C,^15^N]serine labelled U266 cells from ^1^H-^13^C-HSQCs. The serine C2 resonance is predominantly a doublet of doublets of doublets because of *J*_C1C2_ coupling (53.1 Hz), *J*_C2C3_ coupling (36.9 Hz) and *J*_C2N2_ coupling (5.9 Hz). The C2 resonance of creatine is a doublet of doublets as the glycine-derived moiety gives rise to a *J*_C1C2_ coupling (54.5 Hz) and *J*_C2N2_ coupling (11.8 Hz).

Diverse insights into different metabolic pathways are provided by using different labelled precursors as exemplified below:

- Using [3-^13^C]pyruvate as a labelled precursor unexpectedly produced [3-^13^C]serine in both GLOR and U266 cell-lines, although in different amounts (Fig. 6c) providing unique information about gluconeogenesis arising from the activity of pyruvate carboxylase and PEP carboxykinase which convert pyruvate to phosphenolpyruvate, which is then converted to 3-phosphoglycerate and subsequently to serine. PEP carboxykinase is a gluconeogenesis enzyme that was not expected to be active in either of these hematological cancer cell lines.
- Acetate is one of the most rapidly metabolized precursors with detectable label incorporation into glutamate in Kasumi cells after only 5 minutes (Fig. 6d) and may therefore even be suitable for DNP-MRI.
- Using [U-^13^C,^15^N]serine as precursor in the hematological cell-lines, AXP was labelled at the C8 of the adenine ring (Fig. 6e), which is derived from formyl-tetrahydrofolate, illustrating the use of serine in supplying one-carbon metabolism. Purines and pyrimidines represent excellent reporter molecules in nucleobases and when ligated to riboses in nucleotides.
- Using [U-^13^C,^15^N]serine as precursor also led to labelling of creatine (Fig. 7), whose production is altered in many cancers.

### Tracer-based experiments on primary patient tissue

**Figure 8:**
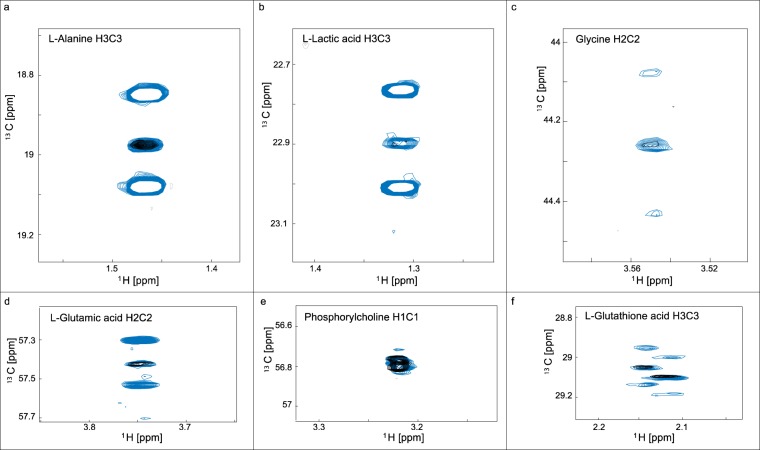
Feasibility of tracer-based analysis from donor liver samples. Overlaid sections from ^1^H-^13^C -HSQC of polar extracts from hepatocytes isolated from a donor liver sample. Black and blue resonances arise from samples exposed for 4.5h to media containing unlabeled glucose and [U-^13^C]glucose, respectively. Panels show the spectral regions for L-alanine C3 (**a**), L-lactic acid C3 (**b**), L-glycine C2 (**c**), L-glutamic acid C3 (**d**), phosphorylcholine C1 (**e**) and L-glutathione C3 (**f**). Phosphorylcholine was not enriched and is included as a scaling reference but the other metabolites were enriched by [U-^13^C]Glucose.

Tracer-based metabolism in primary liver samples has great potential for diagnostic purposes in translational medicine, particularly in the context of understanding glucose and lipid homeostasis in fatty liver disease. Primary human hepatocytes were prepared from liver tissue, as previously described^32^. The harvested primary hepatocytes were metabolically active allowing metabolic studies to measure label incorporations. This is demonstrated in Fig. 8 for a flux time of 4.5 hours with [U-^13^C]glucose. Here we observe label incorporation in alanine, lactic acid, glutamic acid and glutathione. This is in partial agreement with Winnike *et al*.^33^ although their analysis was limited to 1D-^1^H-NMR spectra.

## Discussion

Although NMR has been employed for tracer-based metabolic studies since the 1970s, it is only recent developments in NMR technology that provide the sensitivity required to analyze extracts from mammalian cells. Tracer-based analyses offers considerable opportunities in the context of metabolism in various diseases including cancer. Different NMR approaches include ^13^C-observed spectra, 1D-^1^H spectra where signals arising from ^13^C-coupled protons can be detected, and 2D-^1^H,^1^H-TOCSY spectra. Observing ^13^C directly is very insensitive, even when using ^13^C-observe probes, although the recent development of a much more sensitive ^13^C-observe micro-cryoprobe, which uses high-temperature superconducting receiver coils, may provide additional advantages by observing ^13^C-couplings in the directly observed dimension^34^. The use of proton observed 1D spectra is prone to large errors arising from signal overlap and differences between samples. Indeed errors reported in a recent publication were as large as 20–30%^35^. This is further complicated by the complex line shapes arising from proton-proton and longer-range proton-carbon couplings.

TOCSY spectra have helped to overcome some of these problems (Supplementary Fig. 3) and have been used widely for metabolic analyses^26,36^. One big disadvantage of TOCSY spectra is the long acquisition times, in particular for increased resolution, which are needed to resolve overlapping metabolite signals. This can potentially be overcome with fast TOCSY methods^37^.

Here we propose to base analysis mainly on HSQC spectra and describe a framework to work with reasonable numbers of mammalian cells. For such analyses it is imperative to use the most sensitive probes, at this stage the best option is the micro-cryoprobe available from Bruker. Here we have used a commercially available micro-cryoprobe at 14.1 Tesla (600 MHz proton frequency). We expect further improvements from such probe developments using high-temperature superconducting receiver coils^34^ and by using such probes at much higher field strengths, especially as 28.2 Tesla magnets are on the horizon^38^.

Tracer-based metabolism has started to make an impact in the analysis of metabolic pathways, some of which can only be probed using isotope-labelled tracers. This approach is relevant for future drug discovery in the field of metabolism, and also for elucidating the mechanistic differences between healthy and diseased states in cell lines and, as demonstrated here, potentially also in patient-derived tissue. There have already been reports showing the effectiveness of tracer-based metabolism *in vivo*^39^. In particular, Mashimo *et al*. showed the simultaneous oxidation of [1,2-^13^C]acetate and [1,6-^13^C]glucose in diverse brain tumours^31^. This illustrated the power of using multiple labelled precursors which give distinct labelling patterns in target metabolites. Likewise, [1,2-^13^C]glucose and [3-^13^C]glutamine have the potential to determine the balance between glycolysis and glutaminolysis in supplying the TCA cycle. There is great potential for *in vivo* approaches in metabolically active organs such as the liver and kidney. Such tracer-based studies can be closely linked to DNP enhanced chemical shift MR imaging which can observe the same type of reactions *in vivo*^40,41^, although only within a shorter time frame.

In summary, we present a framework for tracer-based analysis of mammalian cells using an NMR approach that provides robust data for site-specific label incorporations. This type of data can be used to elucidate metabolic mechanisms. With serial sampling, NMR should well complement the well-established MS for metabolic flux analyses. This approach shows that NMR is now suitable for working with limited numbers of mammalian cells.

## Materials and Methods

### Cell cultures

#### Detailed extraction protocol for adherent HuH7 cells

Cell culture: The hepatocellular carcinoma cell line, HuH7, was available in house. Cells were cultured in Dulbecco’s Modified Eagle Medium (DMEM) supplemented with 1% penicillin-streptomycin and 10% (v/v) heat-inactivated fetal bovine serum (FBS), then grown in a humidified atmosphere of 5% CO_2_ at 37 °C in 150 cm^2^ Corning flasks. The cells were routinely subcultured every 3 days (when they reached 80% confluency) by using trypsin to detach the cells from the flask. Briefly, the spent media was discarded, the flask was washed with 10 ml phosphate-buffered saline (PBS) and the PBS was discarded. Then, 5 ml of trypsin was added to the flask and incubated for 5 minutes. After microscopic assessment to confirm the cells were detached, 5 ml of media was added to the flask to inactivate the trypsin. In triplicate, 2 ml of this mixture was then inoculated into 18 ml of media and the flasks were incubated again at 37 °C.

Isotope Labelling: For tracer-based metabolic analysis the cells were transferred to a modified DMEM media lacking the metabolic precursor that was intended to be labelled, and completed with labelled precursors. The cells were then cultured with tracer for a further 6 hours prior to intracellular metabolite extraction. This was sufficient time to give an average cell yield of 15 × 10^6^ cells/ml, the required cell density to provide a strong signal for NMR analysis.

Intracellular Metabolite extraction: PBS and purified water were kept at −4 °C, and the methanol and chloroform at −20 °C prior to use. For the quenching process, the spent medium was aspirated and each flask was washed twice with cold phosphate-buffered saline (PBS). Then, intracellular aqueous metabolites and lipids were extracted using a dual phase extraction procedure adapted from Teng *et al*.^42^. Briefly, 1200 µ of cold methanol (HPLC grade) was added to stop metabolic activity, and the cells were scraped off the flask. Afterwards, the suspension was transferred into a labelled vial and vortexed for 30 s. Chloroform (400 µl + 400 µL) and purified water (600 µl) were then added to each sample, each addition being followed by 30 s vortexing, and the samples were left to rest on ice for 10 min. After centrifuging at 4000 RPM for 10 min, the upper aqueous phase (~500 µ) and the lower organic phase (~200 µl) were then carefully transferred into new vials. The upper (polar) phase was dried under vacuum while the lower organic phase was stored at −80 °C until further analysis.

#### Detailed extraction protocol for haematological cancer cells grown in suspension

Cell culture: Cells were cultured in Roswell Park Memorial Institute (RPMI) 1640 media supplemented with 1% penicillin-streptomycin and 10% (v/v) heat-inactivated fetal bovine serum at 37 °C. Cells were maintained in an exponential proliferation at density 0.5 × 10^6^ cells/ml.

Isotope Labelling: After cell counting, 15 × 10^6^ cells were transferred to a universal tube and centrifuged at 1500 rpm for 5 min at room temperature. The supernatant was discarded and the cells were resuspended in 15 ml media prepared with labelled components. After 6 hours incubation, cells were counted and 10 × 10^6^ cells were centrifuged at 1500 rpm for 5 min at room temperature. 5 ml of the supernatant (media) was taken for media analysis and the remaining media was discarded.

Intracellular metabolite extraction: Methanol was pre-chilled on dry ice and MilliQ water and chloroform were pre-chilled on wet ice. Cells were washed with 1 ml PBS: The cells were transferred to glass vials in 1 ml PBS and then centrifuged at 14000 rpm for 10 seconds. The use of glass vials is essential as chloroform will extract small molecules from most plastic materials. The PBS was removed and 400 μl methanol pre-chilled on dry ice was added. The cell pellet was resuspended thoroughly by vortexing for 10 secs and transferred to a glass vial. After adding 325 μl H_2_O and 400 μl chloroform, the sample was vortexed for 40 secs. The mixture was placed on ice for 10 mins to allow phase separation and then the vials were centrifuged at 4000 rpm for 10 mins at 4 °C in a swingout rotor. After allowing the vials to stand at room temperature for 5 mins, 400μl of the upper (polar) layer and 200μl of the lower (non-polar) layer were transferred to glass vials. Care was taken throughout to avoid carryover of proteins. The upper (polar) phase was dried under vacuum while the lower organic phase was stored at −80 °C until further analysis.

Preparation of primary hepatocytes: Hepatocytes were isolated from marginal human donor livers unsuitable for transplantation surgery under an ethically approved study (Local Research Ethics Committee: reference number 06/Q702/61), with informed patient consent. An encapsulated wedge of tissue was flushed with PBS to remove blood and and initial dissociation buffer was used to loosen cell junctions (10 mM HEPES, 0.5 mM EGTA). After another wash with PBS, tissue was dissociated using a combined enzymatic digestion buffer (0.5% w/v Collagenase A, 0.25% w/v Protease, 0.125% w/v Hyaluronidase and 0.05% w/v Deoxyribonuclease units/mg) as previously described^32^. Following this the liver was mechanically disaggregated in media (DMEM + 10%FCS). The cell suspension was sieved and centrifuged at 50 g to pellet hepatocytes. These were counted using trypan blue and plated out onto collagen-coated plastic.

### NMR spectra

#### NMR sample preparation

Polar extracts were resuspended in 50 µl metabolomics buffer (100 mM sodium phosphate buffer, 0.5 mM TMSP, 10% or 100% D_2_O, pH 7) with vortexing. For samples in 10% D_2_O, 40 µl of supernatant was transferred to champagne vials. 35 µl supernatant was then transferred to 1.7 mm NMR tubes using a Bruker Biospin liquid handler. For samples in 100% D_2_O buffer, 35 µl supernatant was transferred manually to 1.7 mm NMR tubes. Samples were kept at 4 °C prior to NMR.

#### NMR data acquisition

All spectra were acquired at 300 K on a Bruker 600 MHz spectrometer with a TCI 1.7 mm z-PFG cryogenic probe using a cooled Bruker SampleJet autosampler. In all experiments, the ^1^H carrier was on the water frequency and the ^1^H 90° pulse was calibrated at a power of 0.256 W.

For the ^1^H 1D spectra, the standard Bruker pulse sequence noesygppr1d for a 1D NOESY with water pre-saturation was used. Key parameters were as follows: spectral width 11.97ppm/7183.9 Hz; complex points, TD 32768; interscan delay, d1 4 s; short NOE mixing time, d8 10 ms; number of scans, ns = 128; dummy scans, ds = 8. Total experiment time was 14 minutes.

For ^1^H,^13^C-HSQC spectra in water, the pulse sequence used was based on the Bruker standard pulse program hsqcetgpsp, which uses echo/anti-echo TPPI gradient selection. For the ^1^H,^13^C-HSQC spectra in D_2_O, the pulse sequence used was based on the Bruker standard pulse program, hsqcgphprsp. In both cases additional gradient pulses were added to improve water suppression in H_2_O and to help suppress *T*_1_ noise in D_2_O and soft 180°-pulses were used for ^13^C. For the ^1^H observe dimension, a spectral width of 7812.5 Hz/13.02ppm with 1024 complex points was used. For the ^13^C indirect dimension, 2048 complex points were acquired with a spectral width of 24154.6/160.1ppm. Spectra were acquired with 2 scans and an interscan delay of 1.5 s giving a total experiment time of *ca*. 4 h. All spectra were processed using NMRLab in MATLAB (The Mathworks Inc). Cosine-squared window functions were applied to both dimensions. For the NUS spectra, the 25% NUS list used was calculated for 8192 complex points using Wagner’s schedule generator^43^ with a tolerance of 0.01 and other parameters with default values.

#### NMR data processing

1D ^1^H NMR spectra were processed using the NMRlab and Metabolab programs within MATLAB. The free induction decay (FID) signals were multiplied by a 0.3 Hz exponential window function and zero-filled to 32 K points prior to Fourier transformation. Spectra were phased, referenced to TMSP δ 0.00 ppm, baseline corrected and the water region and regions devoid of signal at the edges of the spectrum were excluded. Finally, the spectra were scaled to a constant total spectral area (TSA-scaling). Resonances were assigned using Chenomx (Alberta, Canada, 2015), and by consulting the NMR metabolic profiling human metabolome database (HMDB).

2D HSQC spectra were processed using NMRLab in MATLAB. Cosine-squared window functions were applied to both dimensions and spectra were phased manually. Calibration was carried out manually using L-lactic acid as a reference peak (δ 1.31/22.9 ppm). Peak identification used MetaboLab^24^ with reference to HMDB. For 2D NUS-HSQC, data processing was initially performed using NMRPipe^44^ with the Hyberts extension for processing NUS spectra^43^ and subsequent analysis was then performed with NMRLab as described above for regular HSQC spectra.

#### Intensity scaling of HSQC spectra

To directly derive concentrations from HSQC spectra one needs to consider that *J*_CHx_ coupling constants vary between different compounds. For echo-anti-echo HSQC sequences using a refocussed INEPT (hsqcetgpsp) a complex transfer function is obtained which also depends on the multiplicity x of the CHx group. This becomes considerably simpler for an HSQC that does not use a refocussed INEPT. Nevertheless, the intensity depends on the transfer function. If one wants to derive absolute concentrations from HSQC spectra one has to calibrate for each spin system unless the exact *J*_CHx_ coupling constant is known.

Here we chose not to derive exact concentrations, as this would also require the use of longer recycling times, and relative intensities between labelled and unlabelled, or between different labelled compounds will not be affected by either the transfer function, or *T*_1_ relaxation effects. Moreover, the use of *J*_CC_ couplings is also independent of these factors.

Relative intensities were processed as follows. Theoretically, the percentage label incorporation can be calculated from: I_lab_/I_ctl_ where I_lab_ is the intensity of the resonance from the labeled sample, and I_ctl_ is the intensity of the resonance from the reference unlabeled sample.

It is often impossible to prepare differently labelled but otherwise identical samples because the overall amount of sample depends on cell number. Using a chemical shift referencing standard (usually TMS, TMSP or DSS) is completely unsuitable for intensity referencing because it is added after the sample has been prepared. Therefore, we propose another method to scale HSQC spectra. A common approach to scale one-dimensional spectra is to calculate the total spectral area (TSA) which reflects the total metabolite concentration. The same approach is not possible for HSQC spectra as intensities depend on concentration and label incorporation. However, we can use TSA scaling factors from the one-dimensional spectra to scale the associated HSQCs. Therefore, the expression for the percentage label incorporation becomes (Ilab * Sctl)/(Ictl * Slab), where S_ctl_ and S_lab_ are the scaling factors in the corresponding 1D NOESY spectra for the control and labelled samples. This approach has the advantage that the correction factor uses all the metabolite intensities rather than just one reference metabolite. This method was used to compare intensities between HSQC spectra to obtain label incorporations.

## Supplementary information

Supplementary information
