# Supplementary material for: A framework for tracer-based metabolism in mammalian cells by NMR
Source: Sci Rep. 2019 Feb 21;9:2520. doi: 10.1038/s41598-018-37525-3 (PMC6385278; doi:10.1038/s41598-018-37525-3)
Supplement: Supplementary file 1 — Supplementary information [file 41598_2018_37525_MOESM1_ESM.pdf]

A framework for tracer-based metabolism in mammalian cells by NMR

Raquel Saborano, Zuhal Eraslan, Jennie Roberts, Farhat L. Khanim, Patricia F. Lalor, Michelle AC Reed, Ulrich L Günther

Supplementary Information

Supplementary Figure 1: Using 1D and TOCSY spectra for tracer-based metabolism.

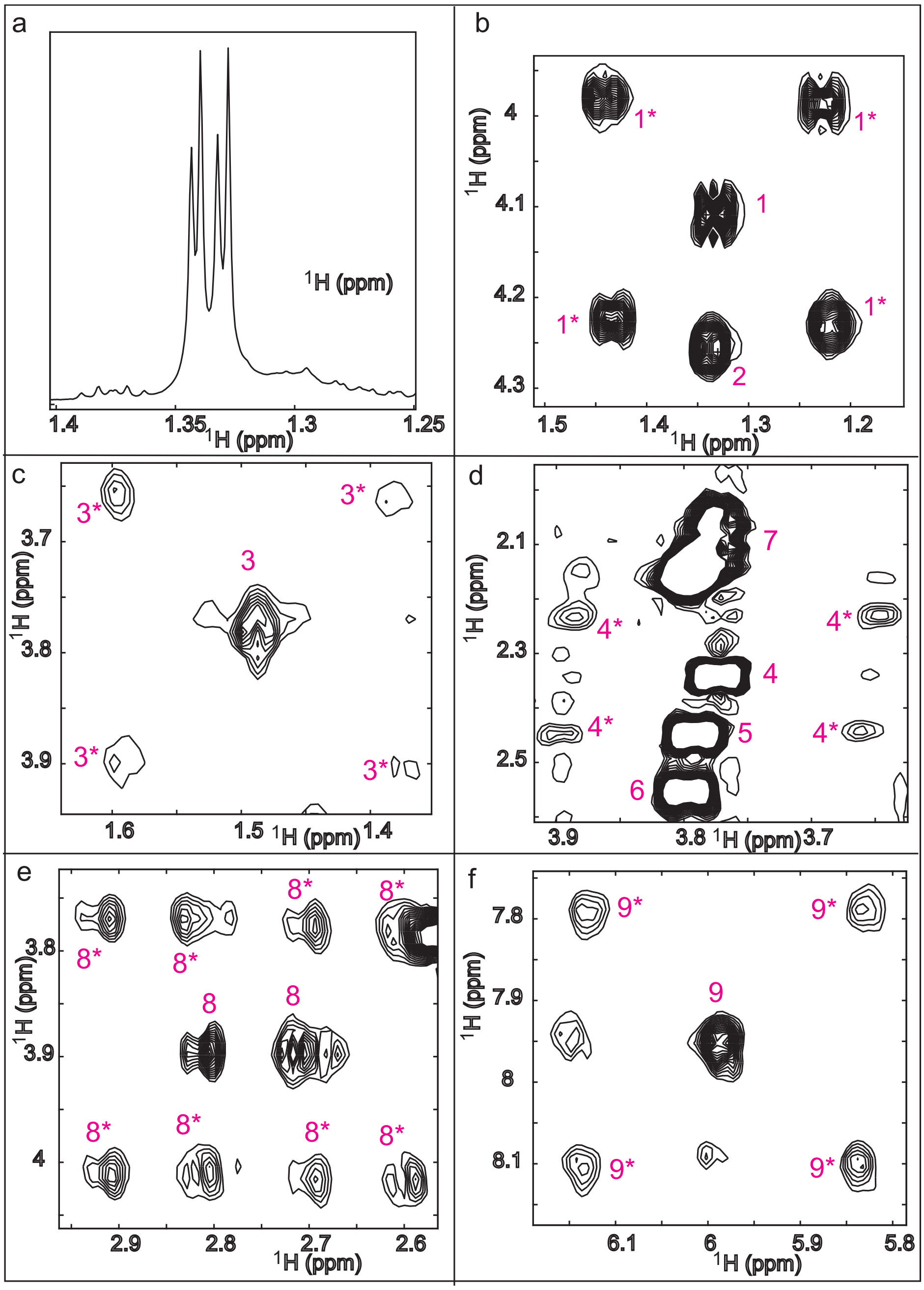

1D and TOCSY spectra have previously been used to analyse labelled metabolite extracts from cells[1] by Lane and Fan, who also list the metabolites and pathways probed. The use of TOCSY spectra is somewhat limited by overlap. While this can be reduced by decoupling ^{13}C in the incremented dimension, we still find this to be a limiting factor. Here, we show some examples where TOCSY spectra can be used. These include lactic acid, threonine, alanine, glutamic acid and glutamine as well as aspartic acid. Panel a shows the region of the ^{1}H NOESY spectrum with overlapped doublets from threonine H4 and lactic acid H3. Panels b-f show sections from the $^{1}H,^{1}H$-TOCSY; 1=lactic acid H3,H2; 2=threonine H4,H3; 3=alanine H3,H2; 4=glutamic acid H2,H4; 5=glutamine H2,H4; 6=glutathione H2,H4; 7=gluta* H2,H3; 8=aspartic acid; 9=UDP H5/H6; * denotes ^{1}H with attached ^{13}C. gluta* is glutathione/glutamine/glutamate.

Supplementary Figure 2:

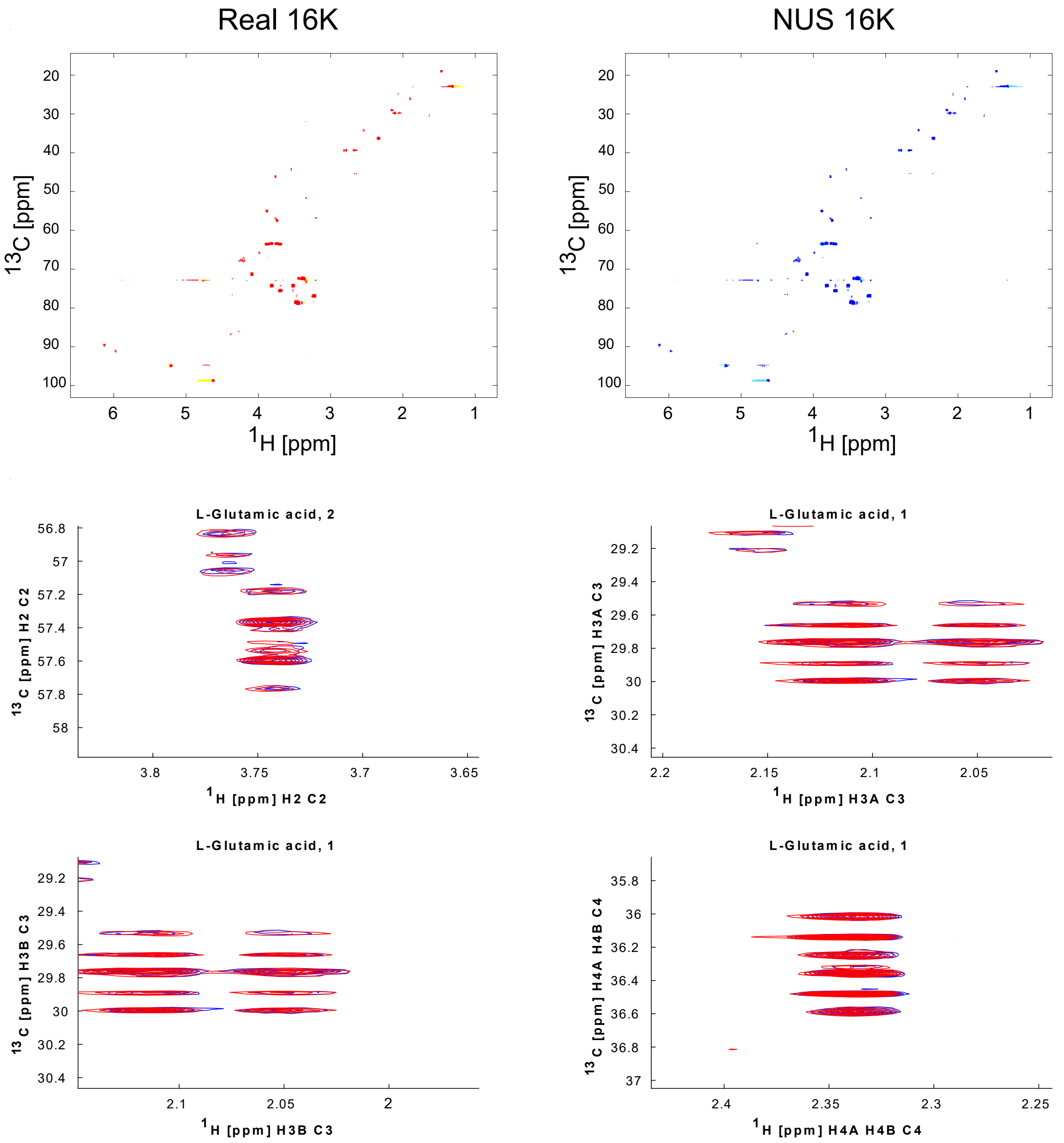

HSQC data for ^{1}H-^{13}C-HSQC spectrum acquired with 16k increments (red) vs 4k increments (blue) with a 25% non-uniformly incremented schedule (NUS). Panel a shows an overview and panel b shows subspectra for glutamic acid. The resulting spectra are virtually indistinguishable; differences are mainly in the noise. NUS spectra were processed using Hybert's[2] hmsIST module in nmrPipe[3].

Supplementary Figure 3:

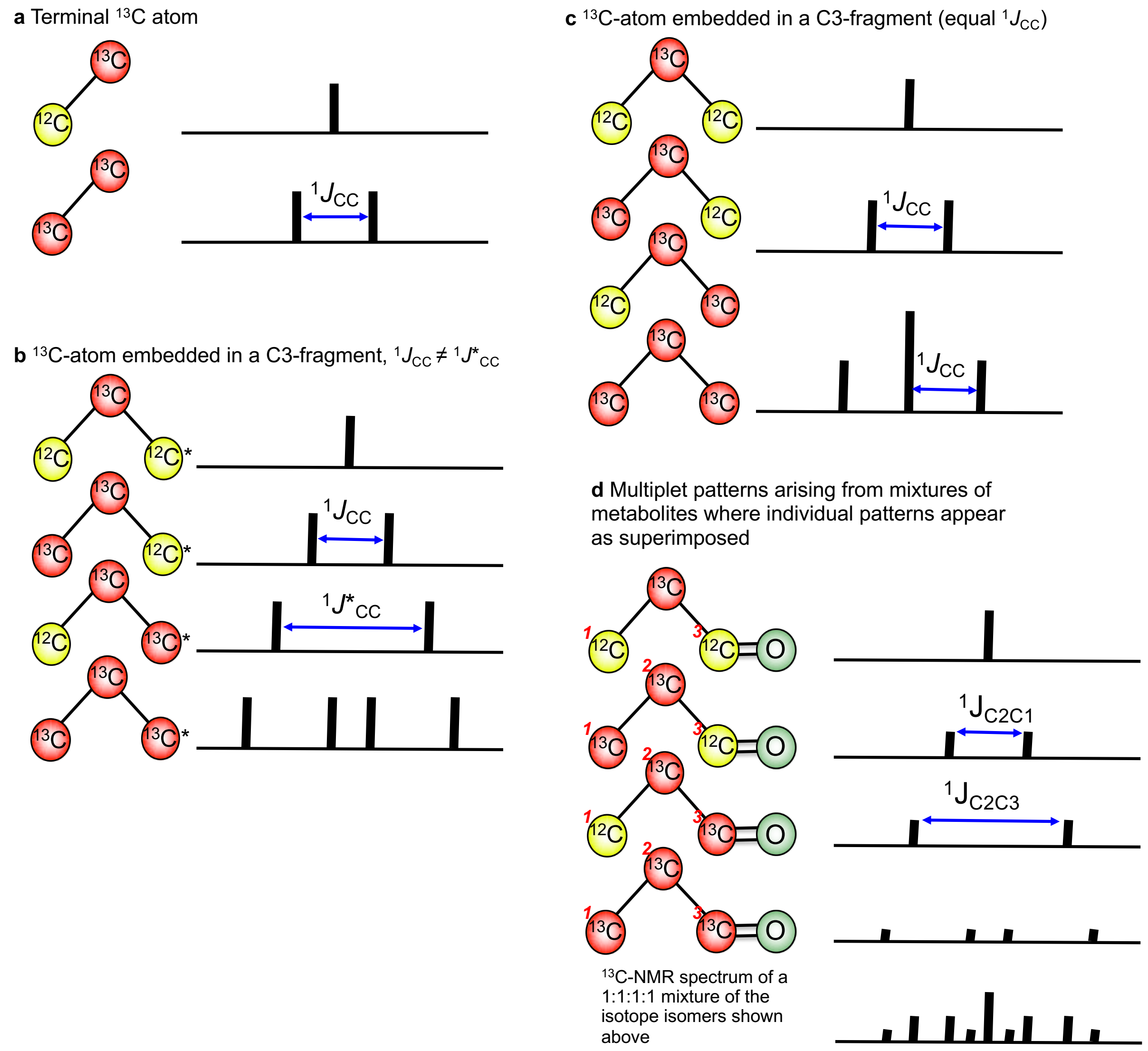

Multiplet structures expected from typical metabolites after ^{13}C-labeling (derived from [4]). Typically observed scalar coupling constants for metabolites are 46-48 Hz for CH_X – CH_X couplings and 50–60 Hz for CH_X–COOH couplings.

Supplementary Figure 4: Label incorporation in purines and pyrimidines

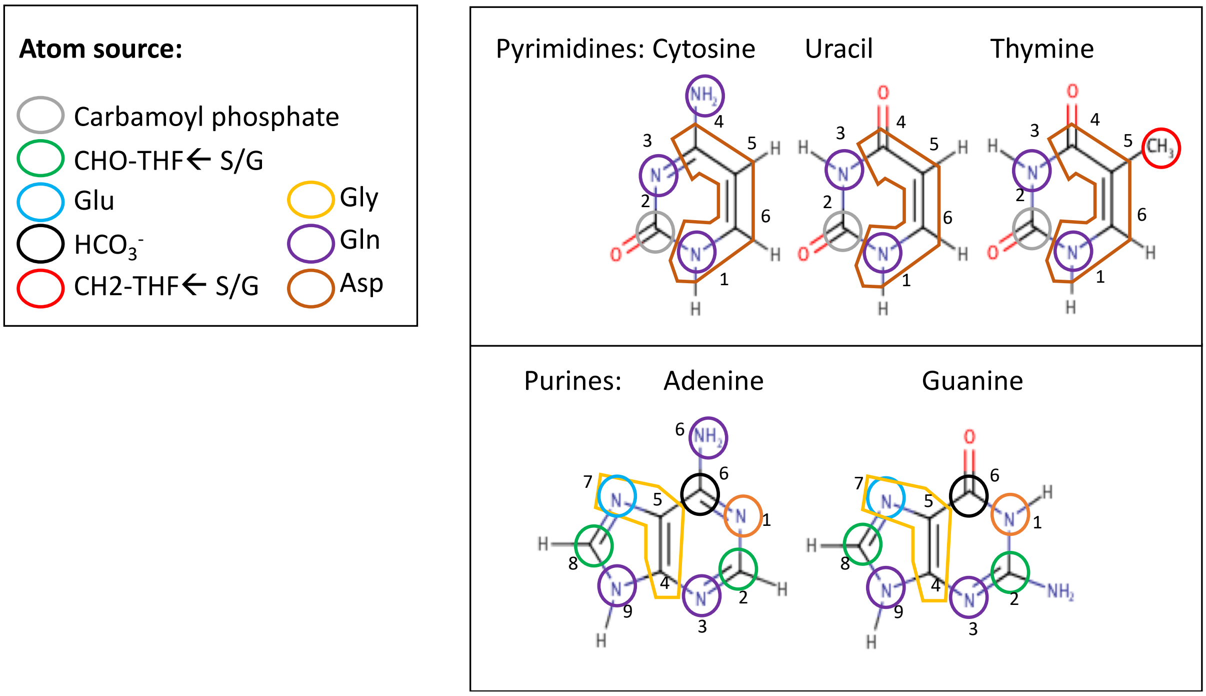

Supplementary Table 1: Metabolic pathways amenable to tracer-based analyses

Pathway	Label & Experiment	Focus	Measurement
Activity of the full Pentose Phosphate Pathway (PPP) vs glycolysis	[1,2-^{13}C]glucose; ^{1}H,^{13}C-HSQC	Lactic acid C3/H3 resonance	The ratio of isolated 3-^{13}C to 2,3-^{13}C-moiety in lactic acid indicates how active the full PPP is compared with glycolysis [1].
Oxidative vs non-oxidative PPP	[1,2-^{13}C]glucose; ^{1}H,^{13}C-HSQC	C1 resonances of riboses	A high ratio of singlet to doublet at C1 is indicative of active oxidative PPP [2].
Glycolysis vs glutaminolysis as inputs to the TCA cycle	[U-^{13}C]glucose (or [1,2-^{13}C]glucose) and [U-^{13}C]glutamine (or [3-^{13}C]glutamine); ^{1}H,^{13}C-HSQC.	TCA cycle intermediates' resonance intensities	Here, the fractional label incorporations into the NMR-observable TCA cycle intermediates (α-ketoglutarate, citrate, fumarate, succinate and malate) and aspartate in samples prepared with labelled glucose and glutamine may be compared [3].
The PC/PDH (pyruvate carboxylase/pyruvate dehydrogenase) ratio	1. [1,2-^{13}C]glucose; ^{1}H/^{13}C-HSQC	1.1 The observed coupling constants at the C2 and C3 of aspartate (& malate)<br><br>1.2 The ratio of the doublet to singlet resonances for the C2-Asp-derived carbon of the pyrimidine base rings.	1.1 PDH gives 1,2- & 3,4-^{13}C-labelled aspartate. These coupling constants are large eg 58Hz. PC gives 2,3-^{13}C-labelled aspartate. The resulting coupling constant is smaller eg 35Hz. The observed coupling constant will lie between these two values unless there is excellent resolution in the ^{13}C-dimension and its exact values changes as the PC/PDH ratio changes.<br><br>1.2 When pyrimidines are synthesized from aspartate, 2,3-^{13}C labelling in aspartate is transferred to C5-C6 in the pyrimidine base ring. However, 3,4-^{13}C labelling in aspartate gives rise to an isolated ^{13}C at C6 as the carbon, C4 of aspartate is lost. Therefore, the ratio of the doublet to singlet resonances at C6 gives an indication of the PC/PDH ratio.
	2. [1,2-^{13}C]glucose; ^{13}C 1D NMR	2. The ratio of doublet to singlet resonance intensities for the carbonate of malonate.	2. PDH gives 1,2-^{13}C- & 3,4-^{13}C-oxaloacetate. This decarboxylates under reactive oxidative stress (ROS) to give a 50:50 mixture of 1,2-^{13}C-malonate and 1-^{13}C-malonate. Three resonances are seen in a ratio 1:2:1 for the carbonate in the ^{13}C spectrum.

			PC gives 2,3-^{13}C-oxaloacetate. This decarboxylates under ROS to give 1,2-^{13}C-malonate and so a doublet is seen for the carbonate in the ^{13}C spectrum [4].
Transamination reactions in nucleotide synthesis	[^{15}N]glutamine; ^{1}H,^{15}N-HSQC optimized for small ^{1}H-^{15}N 2-4J couplings of 10-5Hz.	Resonances in spectra for coupled ^{1}H/^{15}N pairs in purines and pyrimidines	Nitrogens in purine and pyrimidine bases result from transamination reactions using Gln or Glu either directly or indirectly via incorporation of Gly or Asp [5].
γ-glutamyl cycle metabolites	No label; ^{1}H,^{13}C-HSQC.	The C4 resonances of γ-glutamyl cycle intermediates	The C4s of glutamine, glutamic acid, glutathione and pyroglutamic acid are all well-resolved, permitting the identification of changing ratios in response to variables such as drug treatment [6][5].
Creatine production, one carbon metabolism	[U-^{13}C,^{15}N]L-serine	C2 resonances of glycine, guanidoacetate (GAA) and creatine.<br><br>C8 and C14 resonances of AXP	2-^{13}C guanidoacetate is synthesised from arginine and 2-^{13}C glycine. The produced 2-^{13}C guanidoacetate is converted to 2-^{13}C creatine. 8,14-^{13}C AXP results from the synthesis of N^{10}-formyl-tetrahydrofolate and N^5,N^{10}-methenyl-tetrahydrofolate where the transferable carbon is ^{13}C-labelled from one-carbon metabolism.

[1] Glycolysis produces 2,3-^{13}C-lactic acid and unlabeled lactic acid. The full PPP, followed by subsequent glycolysis reactions, and the action of lactic acid dehydrogenase produces unlabeled, 3-^{13}C and 1,3-^{13}C labelled pyruvate and lactic acid molecules as the overall reaction is:

3 x 1,2-^{13}C-Glu6P → 3 x unlabelled G3P + 1 x 3-^{13}C-G3P + 1x 1,3-^{13}C-G3P +3 x ^{13}C-CO_2

where Glu6P is glucose-6-phosphate and G3P is glyceraldehyde-3-phosphate

Thus, when PPP (the oxidative and non-oxidative branch) is upregulated, the intensity ratio of the resonances of lactic acid C3 vs C2 increases, as previously shown by Conti and coworkers[6].

[2] In oxidative PPP, the C1 of glucose is lost as carbon dioxide, generating C5-riboses for nucleotide synthesis. As a result, [1,2-^{13}C]glucose produces [1-^{13}C]riboses. A high ratio of singlet to doublet at C1 is a clear indication of oxidative PPP.

Further reversible reactions of non-oxidative PPP can spread an isolated label to the C2, C3 and C5 positions. As reductive PPP yields [1,2-^{13}C] and [4,5-^{13}C]riboses, the presence of the latter can be taken as proof for reductive PPP, although the overall label distribution is complex considering that many reversible reactions are involved.

[3] For example, in K562, an AML cell-line, fractional label incorporation was approximately five-fold higher from [3-^{13}C]glutamine than from [1,2-^{13}C]glucose suggesting that glutamine is a very important anaplerotic substrate in this cell-line[5].

[4] These approaches to probing PDH vs PC activity were used in our previous work on AML K562 cells. We observed only the doublet for the malonate carbonate in the ^{13}C spectrum of the [1,2-

[13]C]glucose-labelled AML cell extract suggesting high PC activity in this cell-line[7]. Furthermore, we observed a shift from PC to PDH as flux time increased.
In recent spectra using the hsqcphprsp sequence we were able to resolve the PDH and PC products separately (Figure 3e&f).

[5] The following coupled spins can potentially be seen in labelled samples and all were seen with [U-^{15}N]ATP or [U-^{15}N]UTP samples:
In purines:
N1/H2*, N3/H2*, N7/H8, N9/H2*, N9/H8
* adenine only
In pyrimidines: N1/H5*, N1/H6, N3/H5*, N3/H6, N1/H1(of ribose)
* cytosine and uracil only
Source of the atoms in nucleotide bases.

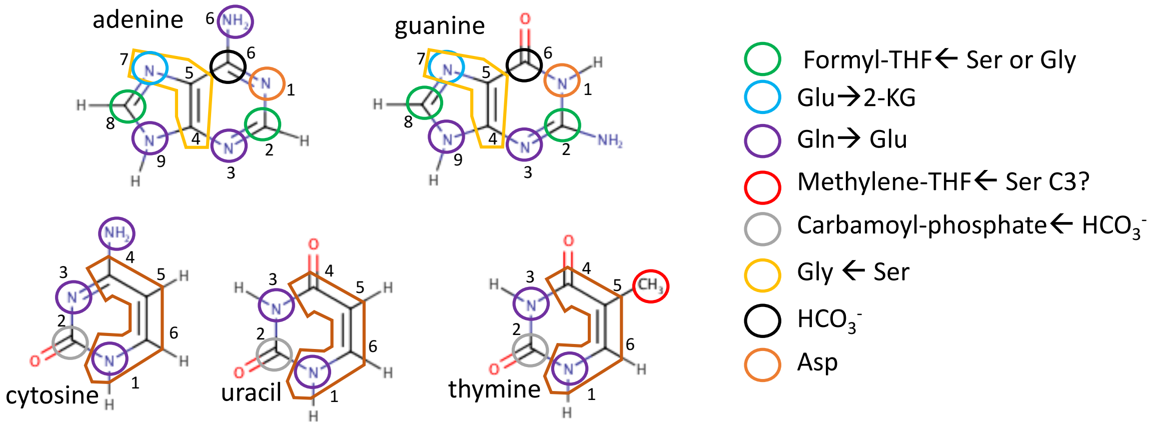

Furthermore, all these resonances were seen in ^{1}H-^{15}N spectra of cell extracts from TALL cell-lines cultured in ^{15}N-glutamine-labelled media except N3/H5 in pyrimidines and N7/H8 in purines.

[6] The γ-glutamyl cycle can be analysed without labeling[5]. This can however not be resolved in 1D spectra. The greater resolution of the 2D spectra was essential for following the changing ratios of the γ–glutamyl cycle intermediates.

References

1. Lane, A. N. & Fan, T. W.-M. NMR-based Stable Isotope Resolved Metabolomics in systems biochemistry. *Archives of Biochemistry and Biophysics* **628,** 123–131 (2017).
2. Hyberts, S. G., Milbradt, A. G., Wagner, A. B., Arthanari, H. & Wagner, G. Application of iterative soft thresholding for fast reconstruction of NMR data non-uniformly sampled with multidimensional Poisson Gap scheduling. *J Biomol NMR* **52,** 315–327 (2012).
3. Delaglio, F. *et al.* NMRPipe: A multidimensional spectral processing system based on UNIX pipes. *J Biomol NMR* **6,** 277–293 (1995).
4. Szyperski, T. Biosynthetically Directed Fractional ^{13}C-labeling of Proteinogenic Amino Acids. *European Journal of Biochemistry* **232,** 433–448 (1995).

5. Carrigan, J. B. *et al.* Tracer-Based Metabolic NMR-Based Flux Analysis in a Leukaemia Cell Line. *ChemPlusChem* n/a-n/a (2016). doi:10.1002/cplu.201500549
6. Miccheli, A. *et al.* Metabolic profiling by 13C-NMR spectroscopy: [1,2-13C2]glucose reveals a heterogeneous metabolism in human leukemia T cells. *Biochimie* **88,** 437–448 (2006).
7. Reed, M. A. C., Ludwig, C., Bunce, C. M., Khanim, F. L. & Günther, U. L. Malonate as a ROS product is associated with pyruvate carboxylase activity in acute myeloid leukaemia cells. *Cancer & Metabolism* **4,** (2016).
